# Two Distinct Neuronal Populations in the Rat Parafascicular Nucleus Oppositely Encode the Engagement in Stimulus-driven Reward-seeking

**DOI:** 10.2174/1570159X22666240131114225

**Published:** 2024-02-01

**Authors:** Mehdi Sicre, Frederic Ambroggi, Julie Meffre

**Affiliations:** 1 Aix-Marseille Université, CNRS, Laboratoire de Neurosciences Cognitives, UMR 7291, Marseille, France;; 2 Institut de Neurosciences de la Timone, Aix-Marseille Univ, CNRS, INT, Marseille, France

**Keywords:** Parafascicular nucleus, thalamic nuclei, PSTHs, neuronal populations, reward-seeking, MOTIV+ neurons

## Abstract

**Background:**

The thalamus is a phylogenetically well-preserved structure. Known to densely contact cortical regions, its role in the transmission of sensory information to the striatal complex has been widely reconsidered in recent years.

**Methods:**

The parafascicular nucleus of the thalamus (Pf) has been implicated in the orientation of attention toward salient sensory stimuli. In a stimulus-driven reward-seeking task, we sought to characterize the electrophysiological activity of Pf neurons in rats.

**Results:**

We observed a predominance of excitatory over inhibitory responses for all events in the task. Neurons responded more strongly to the stimulus compared to lever-pressing and reward collecting, confirming the strong involvement of the Pf in sensory information processing. The use of long sessions allowed us to compare neuronal responses to stimuli between trials when animals were engaged in action and those when they were not. We distinguished two populations of neurons with opposite responses: MOTIV+ neurons responded more intensely to stimuli followed by a behavioral response than those that were not. Conversely, MOTIV- neurons responded more strongly when the animal did not respond to the stimulus. In addition, the latency of excitation of MOTIV- neurons was shorter than that of MOTIV+ neurons.

**Conclusion:**

Through this encoding, the Pf could perform an early selection of environmental stimuli transmitted to the striatum according to motivational level.

## 1 INTRODUCTION

The historic view of the thalamus as a group of nuclei relaying sensory information to the cortex [1] has largely been reevaluated. The thalamus is a phylogenetically ancient structure that evolved before the expansion of the neocortex [2]. Thus, while thalamic nuclei have long been known to provide essential information to the cerebral cortex, it is not surprising that they also intensely connect subcortical regions [3, 4]. In particular, the associative thalamic nuclei from the midline and intralaminar group send strong projections to the striatal complex [5]. This structure is considered the entry station of the basal ganglia, a subcortical network participating in the selection and control of voluntary actions [6, 7]. This pattern of connectivity has challenged a key element of the classical model of the basal ganglia, stating that the input signal to the striatum originates from the cerebral cortex [8]. Some authors even radically proposed that the thalamus, rather than the cortex, may be the dominant source of sensory information to the basal ganglia [9-11]. This appreciation of thalamic afferents to the striatum has largely incentivized their investigation at the cellular and network levels [12-15], and several studies have now begun to address their functional implications at the behavioral level [16-21].

The primate center médian (CM)/parafascicular (Pf) complex within the caudal intralaminar nuclei has attracted a lot of attention. The CM is not present in the rodent brain, but it has been largely documented that the primate CM/Pf complex is equivalent to the rodent Pf [22-24]. There is an extensive topographic projection from the mediolateral axis of the Pf to a ventromedial-dorsolateral axis of the striatal complex in both rats [5, 25, 26] and mice [15]. The medial Pf projects to the ventral region of the striatum (*i.e*., nucleus accumbens, NAc), while the lateral Pf projects to the dorsolateral striatum. A similar topography has also been described in primates [22, 23, 27].

Electrophysiological recordings have shown that primate CM/Pf neurons are rapidly excited by stimuli from a variety of sensory modalities (auditory, visual and somatosensory). These activations depend on the temporal unpredictability of stimuli and their association with rewards [16], suggesting that the CM/Pf is involved in attentional orienting toward salient sensory stimuli [28, 29]. Furthermore, it has been reported that CM/Pf neurons are more excited by stimuli predicting a small reward compared to a large reward [30] and that the magnitude of these excitations inversely correlates with reaction times [31].

Thus, the CM/Pf could provide important information to the striatum to adequately control task engagement in response to temporally unexpected reward-predictive stimuli. To our knowledge, the response of Pf neurons to reward-predictive stimuli has not been investigated in rodents. The aim of this study was to fill this gap by recording single-neuron activity in the rat Pf during a reward-seeking task. In this task, rats were rewarded for performing an instrumental action in response to a predictive stimulus. We used long sessions to compare Pf neuronal responses to stimuli when rats engaged themselves in reward-seeking and responses when they did not. We found a population of neurons that responded more intensely to reward-predicting stimuli when the animals subsequently engaged in the task compared to when they did not. A second population, which responded to stimuli at shorter latency, had an opposite pattern of activity. We propose that these two populations may respectively allow animals to participate in or refrain from active engagement in response to reward-predictive stimuli through their connections to the NAc.

## 2 EXPERIMENTAL PROCEDURES

### 2.1 Subjects

Experiments were conducted on male Long-Evans rats (Charles Rivers, France) weighing ~300 g on arrival. Rats were immediately housed individually on a 12 h light/dark cycle. Experiments were conducted during the light phase. After one week of habituation, rats were placed under food restriction; food rations were adjusted daily to maintain the body weight at ~90% of their free-feeding body weight. All experiments were performed in accordance with the guidelines on animal care and use of the European guidelines (European Community Council Directive, 2010/63/UE) and National guidelines.

### 2.2 Training

All experiments were conducted in operant chambers containing two house lights, a tone speaker, a retractable lever and a reward receptacle located on one wall of the chamber (Med Associates, Vermont, USA). A fan-generated background noise (~60 dB). Liquid sucrose (10%) was delivered as a reward in the receptacle by a syringe pump. During the first 2 days, rats were trained to obtain 50 µl of sucrose by spontaneously entering the reward receptacle. When 300 rewards were obtained within an hour, rats were run on a fixed-ratio 1 (FR1) schedule with a 10 s-time-out: the lever was constantly extended in the chamber, and a lever-press triggered the delivery of 50 µl of sucrose into the receptacle. When rats reached the criterion >100 lever-presses within an hour, they were advanced to the stimulus-driven reward-seeking task.

### 2.3 Stimulus-driven Reward-seeking Task

Rats were run daily on the task for 3 hours. The extension of a lever was used as an auditory/visual instructive incentive stimulus. The lever was extended in the cage in 300 ms *via* a motor that generated a noise. Because the motors generated different noise levels in the 3 recording cages used for this study, we coupled the extension with a 300 ms/85 dB speaker-generated white noise. When the lever was extended, rats had 10 seconds to press it to obtain the reward. Each trial was followed by a variable interval schedule averaging 45 s (from 30 to 60 s). If the rats did not lever-press within 10 s, the lever retracted, and the intertrial interval was re-initialized. Surgery was performed when rats reached the criterion of > 80% responses during the first hour of the session.

## 3 SURGERY

Rats were anesthetized with 5% isoflurane and placed in a stereotaxic apparatus (Kopf Instruments, California, USA). Animals received an injection of buprenorphine (0.05 mg/kg, Vetergesic, France), and the anesthesia level was adjusted with 0.5-2% isoflurane during the maintenance phase. Before skin incision, subcutaneous injection of lidocaine (1mg/ml, Lurocaine MedVet) was performed. Bundles of 8 electrodes were attached to custom-made microdrives [32] that allowed to lower the electrode bundles by 80 µm increments. Electrodes were implanted in the Pf bilaterally in 5 animals and unilaterally in 2 animals (one on the left and the other on the right hemisphere) at the following stereotaxic coordinates: AP: -4.1, ML: +/-1, DV: -5.4 mm relative to the Bregma. The microdrives and the connectors were secured to the skull with bone screws, adhesive cement (C&B Metabond, Phymep, France) and dental acrylic (Phymep, France). After surgery, a prophylactic analgesic treatment (Buprenorphine, 0.05 mg/kg, Vetergesic, France) was administered, and rats were given at least 7 days of recovery with *ad libitum* access to food. After recovery, rats were placed under food restriction and re-trained until reaching the previous criteria.

## 4 ELECTROPHYSIOLOGY

### 4.1 Recording Procedure

Electrophysiological recordings were conducted as described previously [33] during 3 hour-long sessions. Animals were connected to the electrophysiological acquisition system (SpikeGadget LLC, California, USA). The 32-channel head stage, streaming data at 30 kHz per channel, was connected to a low-torque HDMI commutator that allowed the animals to be free of their movements in the chamber. Between sessions, electrode bundles were lowered by 80 or 160 µm increments to record a new set of neurons. Unfiltered data were transferred from the data acquisition main control unit to a data acquisition computer, where it was visualized and saved. Digitally-filtered data (0.2-6 kHz) were used for spike sorting.

### 4.2 Spike Sorting

Recorded data were analyzed with OfflineSorter (Plexon Inc, Texas, USA) to isolate spikes from single neurons with principal component analysis. Inter-spike interval distributions, cross-correlograms and auto-correlograms were used to ensure that the activity of single neurons was isolated. Only well-isolated waveforms with characteristics that were constant over the entire recording session were included in this study. Sorted units were exported to NeuroExplorer 4.135 (Nex Technologies, Colorado, USA) and Matlab R2022b (MatWorks Inc, Massachusetts, USA) for further analysis.

### 4.3 Electrophysiological Analyses

#### 4.3.1 Waveform Analysis

In our data set, 290 neurons showed waveforms with a negative followed by a positive deflection. The remaining 95 neurons displayed the opposite pattern. The spike width was assessed by the time elapsed between the first and second extremum independently from the sign of the first and second deflections. The spike width of 14 neurons could not be determined due to technical issues, and we then excluded them from the analyses involving this parameter.

#### 4.3.2 Responses Detection

Peri-stimulus time histograms (PSTHs) were constructed with smoothed (lowess method, span = 4) 20 ms- and 2 ms-time-bins. PSTHs constructed around the behavioral events (stimulus presentation, lever-press and reward collection) were used to detect excitations and inhibitions and the time at which they occurred. The 10 s period before the presentation of the stimulus was used as a baseline period. Excitation and inhibition to each event were determined by the presence of at least 3 consecutive bins above the 99% (for excitations) or below the 1% (for inhibitions) confidence interval of the baseline during the analysis windows (0 to 250 ms after the stimulus, -2 to 1 s around lever-presses and reward delivery). Onset was determined by the time of the first of 3 consecutive bins falling outside the confidence interval. The offset was determined, in analogy, by searching the first of 6 consecutive bins within the confidence interval.

#### 4.3.3 Deconvolution

To isolate the activity of temporally close events, we used a deconvolution method as described previously [34, 35]. Briefly, the model assumes that the total firing rate of a neuron in each trial is equal to the linear sum of the contributions of each event-related firing, delayed by the event latency in that trial. Here, we deconvolved single-event responses for each neuron using the optimal number of iterations that had a cross-validation error lower than the PSTHs.

#### 4.3.4 Data Normalization and Plotting

Color-coded maps and average PSTHs across neurons were constructed with 20 ms- and 2 ms-bins. Prior to averaging, the firing rate of each neuron during each bin was z-score-transformed: (F_i_ - F_mean_)/F_sd_ where F_i_ is the firing rate of the i^th^ bin of the PSTH, and F_mean_ and F_sd_ are, respectively, the mean and the SD of the firing rate during the 10 s preceding stimulus event onset.

### 4.4 Statistical Analyses

We sought to compare the trials to which the animal engaged in reward-seeking with those it did not. Thus, we selected the sessions containing at least 20 trials of each type to conduct a reliable analysis of the neuronal data. For behavioral analyses, the primary dependent variables were the percentage of responding to the stimulus and the latency to lever-press after the presentation of the stimulus.

For electrophysiological data, the primary dependent variables were the baseline firing rate, the maximal frequency reached (measured by averaging the top 5% of instantaneous frequencies during the baseline period), the coefficient of variation (measured during the baseline period), the spike width, the proportion of responsive neurons, the mean z-score normalized firing (0-250 ms post-stimulus and -2 to 1 s around lever-presses and reward collection), the onset latency and the durations of the responses. These variables were analyzed with paired, unpaired t-tests or ANOVAs. When appropriate, a Bonferroni test was used to conduct post-hoc comparisons. Proportions were analyzed with χ^2^ tests and distributions with Kolmogorov-Smirnov tests. All results were considered significant at *p* < 0.05. Data are presented as mean+/-SEM.

### 4.5 Histology

Animals were euthanized, and each electrode site was labeled by passing a 20 µA DC current for 7 s. Rats were perfused intracardially with phosphate-buffered saline followed by a 10% formalin solution. Brains were removed, post-fixed in 10% formalin and placed in 30% sucrose for 3 days. Brains were sectioned at 40 µm on a cryostat, and slices were stained with cresyl violet. Reconstruction of the recording sites was made based on the final location of the electrodes.

## 5 RESULTS

### 5.1 Behavioral Analysis

**Fig. (1):**
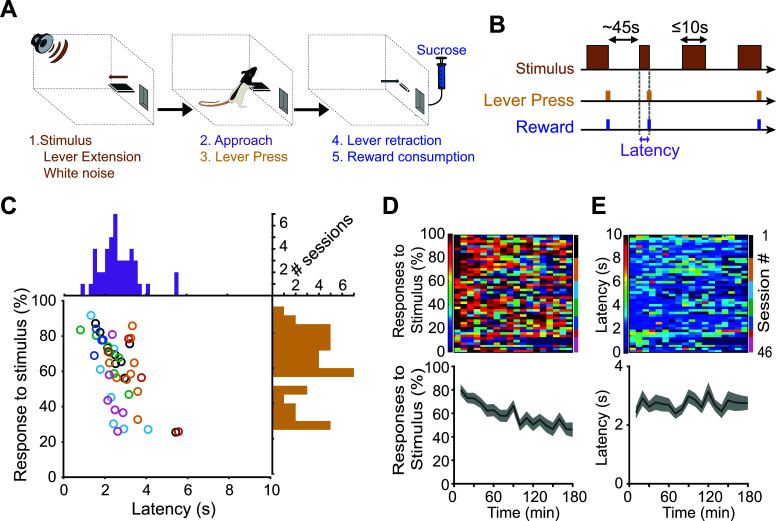
Behavioral performance in the stimulus-driven reward-seeking task. (**A**) Task diagram showing the sequence of events. (**B**) Temporal structure of the task. (**C**) Individual percentage of responses and latencies for all sessions (n = 46). Animals (n = 7) are color-coded. Histograms represent the distributions of the percent responses (yellow) and latencies (purple). (**D**) Top: Heatmaps representing the percentage of responses to the stimulus over time. Each line corresponds to a single session. Data are grouped per animal (labeled with the right color bar). Bottom: average percent responding as mean+/-sem. (**E**) Same representation as in D for behavioral latencies.

Rats performed a stimulus-driven task where a compound auditory/visual stimulus was presented unexpectedly (on average every 45 s). The stimulus was a 300 ms-white noise coupled to the extension of a lever lasting up to 10 s (see methods for details). A lever press triggered the immediate delivery of a 10% sucrose reward in an adjacent receptacle and the retraction of the lever (Figs. **1A**, **B**). We sought to analyze neuronal activity when the rats attended to the stimulus and when they did not. In order to obtain a sufficient number of trials of each type, we ran 3-hour long sessions. For all rats, the percentage and latency of lever-pressing in response to the reward-predictive stimulus were variable across sessions (5 to 9 sessions per rat for 7 rats, a total of 46 sessions Fig. **1C**). Overall, the mean response percentage was 60.9 ± 2.8% with an average stimulus-lever-press latency of 2.64 ± 0.14 s. The response percentage decreased over time (F17,773) = 3.92, *P <* 1.9x10^-7^, Fig. **1D**), reflecting a change in motivation as rats accumulated sucrose rewards. However, this effect was not found in the response latency, which remained stable over time (F(17,673) = 0.69, *P =* 0.836, Fig. **1E**).

### 5.2 Basic Electrophysiological Properties of Pf Neurons

**Fig. (2):**
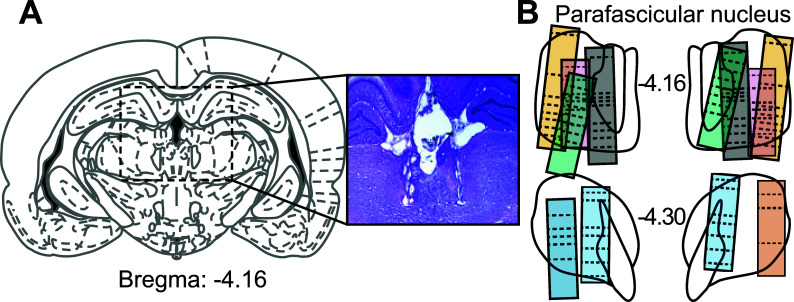
Histology and electrode path reconstruction. (**A**) Schematic diagram of a rat brain coronal section and representative photomicrograph of two electrode bundle tracks located in the Pf. (**B**) Histological reconstruction of the recording sites in the Pf. Boxes represent the approximate extent of the electrode bundles in the 7 animals (color-coded). The dotted line corresponds to the depth of the recording sessions.

**Fig. (3):**
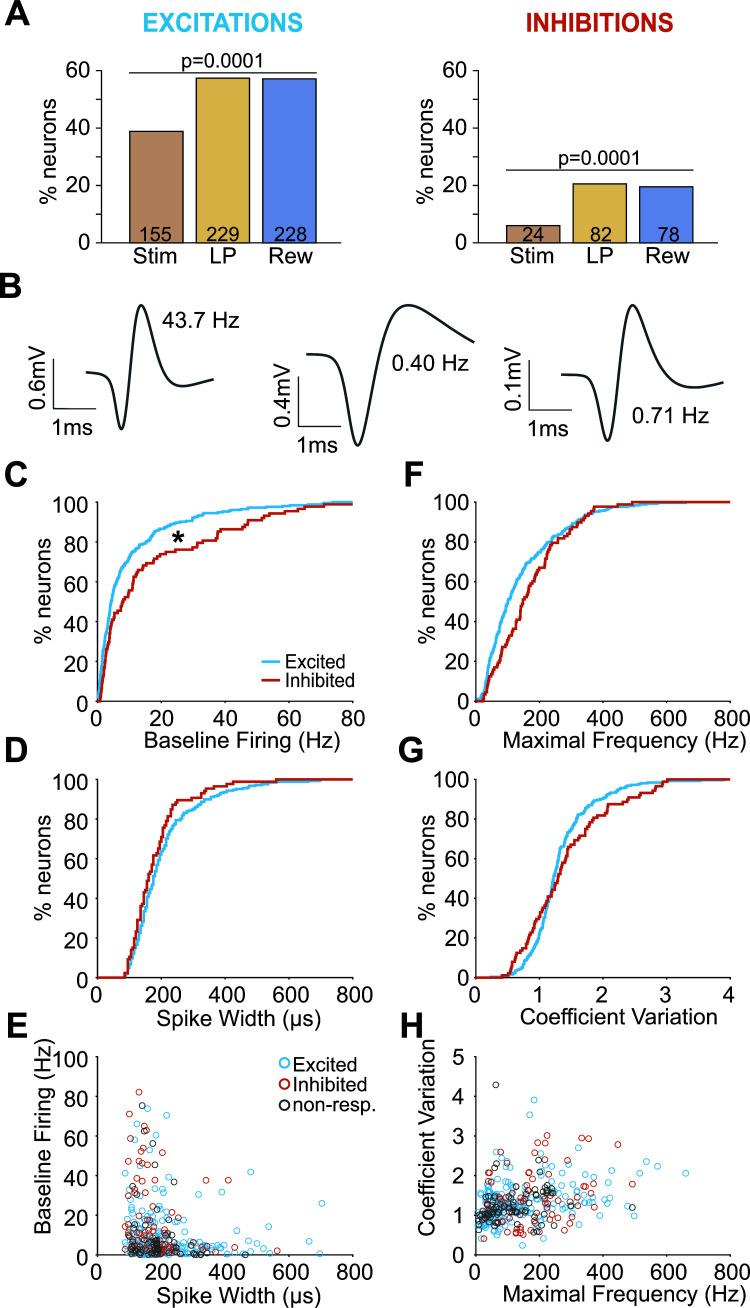
Electrophysiological characteristics of Pf neurons excited and inhibited by the different task events. (**A**) Percentage of neurons excited (*left*) and inhibited (*right*) by the different events (stimulus, lever-press, reward). (**B**) Example of three representative waveforms recorded in the Pf with their baseline firing rate. (**C**) Cumulative percentage of baseline firing rate of task-excited (blue) and task-inhibited (red) neurons. (**D**) Cumulative percentage of spike widths. (**E**) Baseline firing rate plotted against spike width for individual neurons (gray dots indicate non-responsive neurons). (**F**) Cumulative percentage of maximal frequencies. (**G**) Cumulative percentage of the coefficients of variation. (**H**) Coefficient of variations plotted against the maximal frequencies for individual neurons. *P < 0.05.

We recorded the activity of 399 Pf neurons (see Fig. **2** for histological reconstruction of the electrode sites). We first conducted analyses of neuronal responses to different task events: stimulus presentation, lever-pressing and reward collection. For this, we selected the attended trials in which the animals responded to the stimuli by lever-pressing and collecting rewards (Fig. **3**).

We found that the majority of neurons were eventmodulated, and of these neurons, more showed excitatory responses compared to inhibitory ones (254 and 88 neurons, respectively, χ^2^ = 141, *P <* 0.0001). Excitations evoked by the stimulus occurred less frequently than those evoked by the lever presses or the rewards (155, 229 and 228 neurons, respectively, χ^2^ = 27.9, *P <* 0.0001, Fig. **3A**). The different patterns of excitation/inhibition for different events were found in all seven recorded rats (Supplementary Table **S1**).

Across the population, we observed neurons with different waveform profiles and firing properties during the baseline period (*i.e*., 10 s preceding stimulus onset, Fig. **3B**). The combination of responses to the different events was highly variable (Supplementary Table **S2**). As the number of neurons with opposite responses for different events was relatively low (49 out of 399, 12%), we decided to analyze separately the neurons excited and inhibited by at least one task event. To account for rat variability, we included a subject-dependent factor in a two-way ANOVA test. Overall, event-excited neurons had a lower baseline firing rate than event-inhibited neurons (9.81+/-0.78 *vs*. 15.27+/-1.63 Hz respectively; Response direction effect F(1,371) = 4.62, *P =* 0.032, Subject effect F(4,371) = 0.22, *P =* 0.93, Response direction x Subject F(4,371) = 0.19, *P =* 0.94, Fig. **3C**). The other distributions were similar between excited and inhibited neurons (maximal frequency, 137.2+/-6.4 *vs.* 170.3+/-9.1 Hz respectively, Response direction effect F(1,371) = 1.81, *P =* 0.18, Subject effect F(4,371) = 3.97, *P =* 0.004, Response direction x Subject F(4,371) = 0.26, *P =* 0.90; spike width, 206+/-6 *vs*. 175+/-6 ms respectively, Response direction effect F(1,371) = 3.15, *P =* 0.08, Subject effect F(4,371) = 3.99, *P =* 0.003, Response direction x Subject F(4,371) = 0.68, *P =* 0.61, coefficient variation, 1.31+/-0.03 *vs*. 1.36+/-0.05, Response direction effect F(1,371) = 0.51, *P =* 0.47, Subject effect F(4,371) = 5.06, *P =* 0.0005, Response direction x Subject F(4,371) = 0.79, *P =* 0.53, Figs. **3C-G**). When these variables were plotted against each other, event-excited and inhibited neurons strongly overlapped, and the two could not be separated based on these properties (Figs. **3E**, **H**). We conducted similar analyses on excited and inhibited neurons separately for each event and obtained similar results (data not shown). Taken together, these results indicate that even if excited and inhibited neurons were to have different spiking properties, these differences were insufficient to separate the two populations.

### 5.3 Pf neuronal Responses to Task Events

**Fig. (4):**
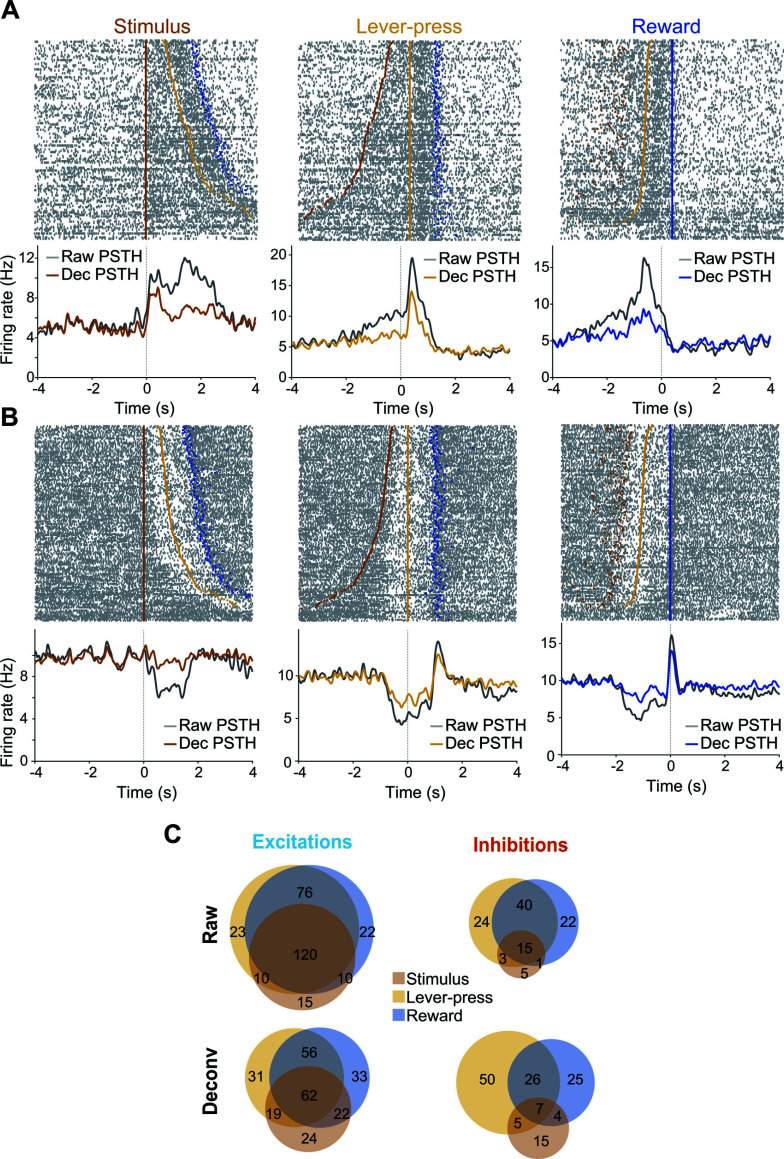
Deconvolution. (**A**) Activity of an example neuron aligned to the stimulus (left), lever press (middle) and reward delivery (right). Top: raster plot with trials sorted by the latency to lever-press. Each gray line is an action potential. Brown squares represent stimuli presentations, orange squares the lever-presses, and blue squares represent reward deliveries. Bottom: Gray lines represent the raw PSTH, showing the average firing rate. Colored lines represent the deconvolved PSTH. (**B**) Same representation for another example neuron. (**C)** Venn diagrams showing the proportion of neurons excited (*left*) and inhibited (*right*) by task events using raw (top) or deconvolved (bottom) data.

We then analyzed the temporal dynamics and magnitudes of these different event-evoked responses. While these neuronal responses often lasted several seconds, the behavioral events that evoked them, stimulus presentation, lever-pressing and reward collection occurred in close temporal proximity with much shorter latencies. PSTHs constructed around each of these events can potentially be distorted by the presence of other temporally correlated events. Thus, PSTHs do not allow one to accurately account for the neuronal responses to each individual event. To circumvent this issue, we used an iterative deconvolution method that takes advantage of the trial-to-trial variability in the temporal relationship between the different events to parse out the neuronal responses of each individual event from the PSTHs [34]. For each neuron, we deconvolved single-event responses by using the optimal number of iterations that achieved a cross-validation error lower than the PSTHs (5.2+/-0.2 iterations for the entire dataset). Fig. (**4A**) shows the rasters and PSTHs of an example neuron aligned to the three events. When the activity was lined up to stimulus presentation (left panel), the PSTH (gray line) showed a bimodal response, with the first activity peak related to the stimuli themselves and the second to lever-presses/rewards that occurred at different latencies on different trials (see raster). Deconvolution (brown line) reduced the contribution of the lever-press/reward-related activity while preserving the initial excitation to the stimulus. When aligned to lever-press, the raw PSTH shows a ramp followed by a sharp peak during the first second after the lever-press. Deconvolution suppressed the ramp that was mainly driven by the stimulus presentations but kept the peak response unaffected. Similarly, the peak preceding reward delivery (right panel) was largely reduced by deconvolution, indicating that this activity was driven by lever-presses rather than reward deliveries. Fig. (**4B**) shows the activity profile of another neuron. The inspection of the rasters shows that this neuron was inhibited before and after lever-press and excited at reward delivery. As in the previous example, deconvolution allowed us to isolate these event-related firing responses by removing the contribution of the adjacent events. Overall, deconvolution significantly reduced the number of responses per neuron (χ^2^ = 44, *P =* 1.4x10^-9^, Fig. **4C**).

**Fig. (5):**
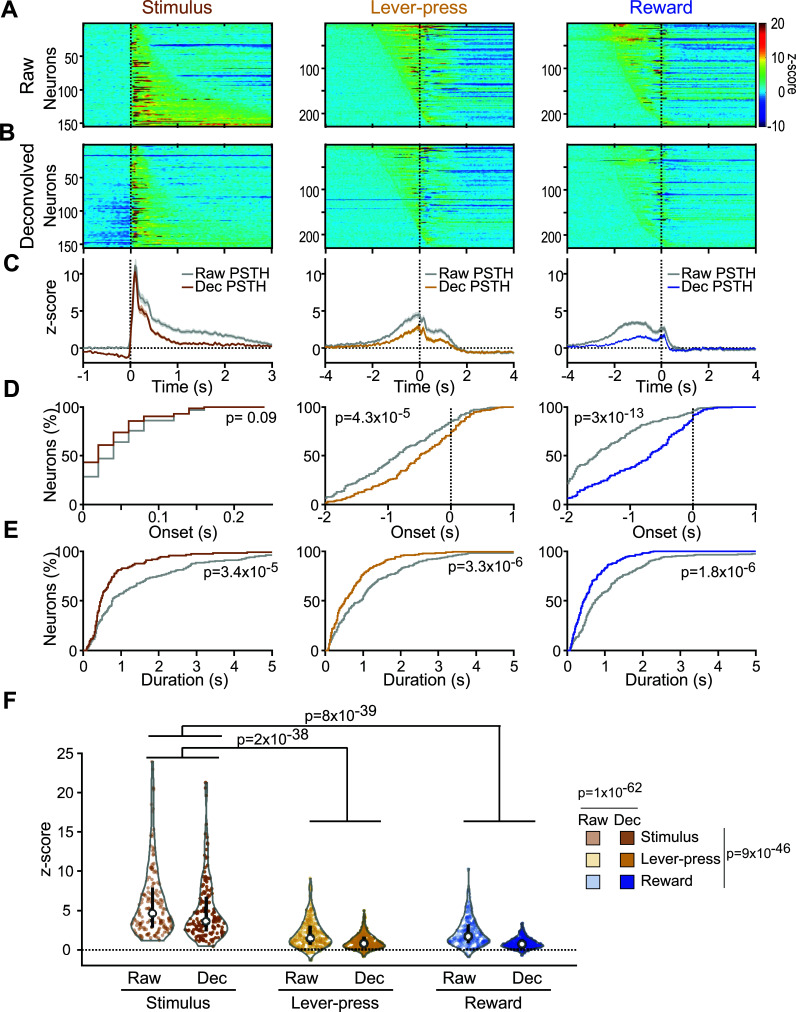
Pf neuronal excitations to task events. (**A**) Heatmaps represent color-coded PSTHs showing neurons excited to the stimulus (*left*), lever-press (*middle*) and reward delivery (*right*). Each row represents the PSTH of an individual neuron aligned to the event considered. Data are plotted with smoothed 20 ms-time bins, and neurons are sorted by excitation durations for stimuli responses and onset latencies for lever-press and reward responses. (**B**) Heatmaps represent deconvolved PSTHs of the same neurons represented in A. (**C**) Average responses for the neurons shown in A and B. The gray traces correspond to raw PSTHs. Brown, orange and blue traces correspond to the deconvolved PSTHs to the stimulus, lever-press and reward delivery, respectively. (**D**) Cumulative percentage of excitation onset latencies for raw and deconvolved responses. (**E**) Cumulative percentage of excitations durations. (**F**) Violin plots of the event-evoked firings. White dots indicate the median and black bars indicate the 25^th^ and 75^th^ percentiles of the distribution. The gray envelope is a rotated kernel density plot, and colored dots show the individual values.

The analysis of raw PSTHs aligned to the stimulus revealed that excitatory responses occurred at a stereotypical latency (58+/-4 ms, Figs. **5A**-**D**) with variable durations (1.57+/-0.17 s, Figs. **5A**, **C**, **E**). The removal of the contribution of neighboring events by deconvolution had no effect on the onset latency distribution of stimulus-evoked excitations (KS = 0.147, *P =* 0.09, Fig. **5D**) but strongly decreased their durations (KS = 0.278, *P =* 3.4x10^-5^, Fig. **5E**).

On raw PSTHs, most lever-press-evoked excitations emerged before the occurrence of the event (-0.830+/-0.05 s) and lasted up to 4 s (1.26+/-0.11 s). Reward-evoked excitations occurred even earlier (-1.36+/-0.04 s) and lasted an average of 1.18+/-0.10s (Fig. **5A**, **C**, **D**). Deconvolution significantly shifted lever-press- and reward-evoked excitations closer to the event (KS = 0.226, *P =* 4.3x10^-5^ and KS = 0.382, *P =* 3.0x10^-13^, respectively, Fig. **5D**) and considerably shortened their durations (KS = 0.25, *P =* 3.3x10^-6^ and KS = 0.26, *P =* 1.8x10^-6^, respectively, Fig. **5E**).

The analysis of the magnitude of evoked responses (Fig. **5F**) revealed a strong influence of the event considered (2-way Repeated Measures ANOVA, Event effect, F(2,606) = 123.6, *P =* 9x10^-46^) and the use of deconvolution (Deconvolution effect, F(1,606) = 355, *P =* 1.1x10^-62^) but no significant interaction between them (Event x Deconvolution, F(2,602) = 2.34, *P =* 0.097). Stimuli-evoked excitations were ~3 times significantly larger than those evoked by lever-presses and rewards (Bonferroni test on Event effect, *P =* 2x10^-38^ and *P =* 8x10^-39^, respectively), but lever-press- and reward-evoked excitations did not differ from each other.

**Fig. (6):**
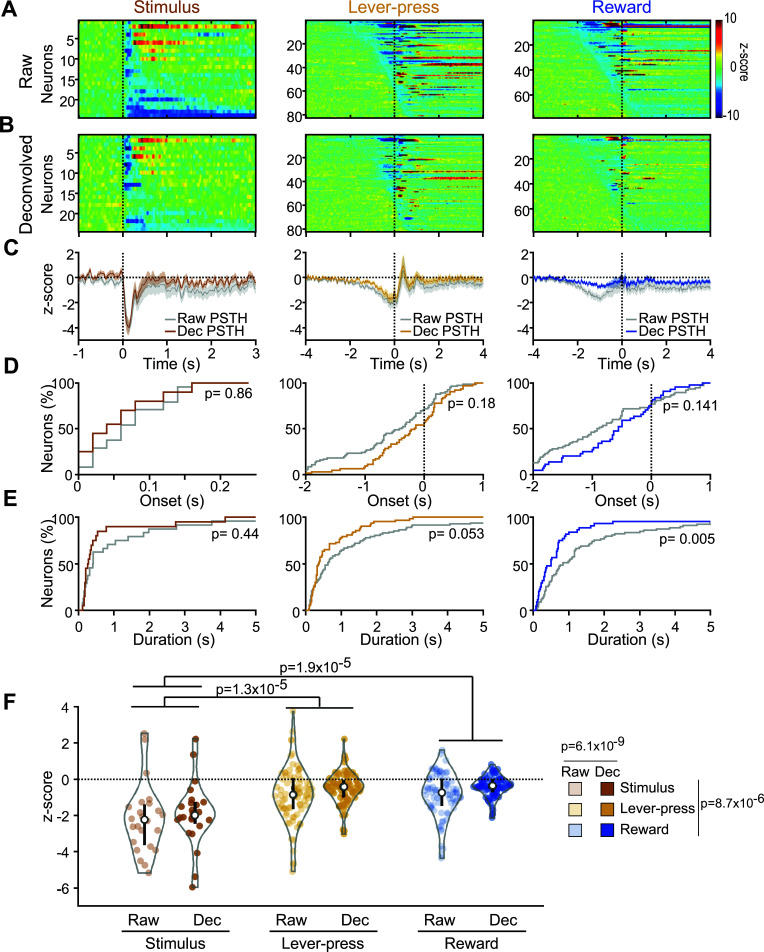
Pf neuronal inhibitions to task events. (**A**) Heatmaps represent color-coded PSTHs showing neurons inhibited to the stimulus (*left*), lever-press (*middle*) and reward delivery (*right*). Each row represents the PSTH of an individual neuron aligned to the event considered. Data are plotted with smoothed 20 ms time bins, and neurons are sorted by inhibition durations for stimuli responses and onset latencies for lever-press and reward responses. (**B**) Heat maps represent deconvolved PSTH of the same neurons represented in A. (**C**) Average responses for the neurons shown in A and B. The gray traces correspond to raw PSTHs. Brown, orange and blue traces correspond to the deconvolved PSTHs to the stimulus, lever-press and reward delivery, respectively. (**D**) Cumulative percentage of inhibition onset latencies for raw and deconvolved responses. (**E**) Cumulative percentage of inhibition durations. (**F**) Violin plots of the event-evoked firings. White dots indicate the median and black bars indicate the 25^th^ and 75^th^ percentiles of the distribution. The gray envelope is a rotated kernel density plot, and colored dots show individual values.

Inhibitions analyzed on raw PSTHs shared many similarities with excitations, but deconvolution had less effect, suggesting that these responses were more temporally associated with the behavioral events (Fig. **6**). Stimulus-evoked inhibitory responses occurred after 85+/-9 ms (non-significantly different from excitations, KS = 0.264, *P =* 0.09, Figs. **6A**, **B**, **D**) and lasted 1.38+/-0.63 s (significantly shorter than excitations, KS = 0.335, *P =* 0.0014, Fig. **6E**). Deconvolution had no effect on these measures (KS = 0.175, *P =* 0.85 and KS = 0.25, *P =* 0.44, respectively, Figs. **6B-E**).

Inhibitions to lever-presses and rewards preceded the events for most neurons (-0.56+/-0.09 s and -0.82+/-0.10, respectively, Figs. **6A**, **C**, **D**) and deconvolution had no effect (KS = 0.18, *P =* 0.18 and KS = 0.21, *P =* 0.14, respectively, Figs. **6B**, **C**, **D**). We observed a strong trend toward a reduction of the duration of lever-press-evoked inhibitions by deconvolution (KS = 0.22, *P =* 0.053). The durations of reward-evoked inhibitions were reduced significantly (KS = 0.312, *P =* 0.006, Fig. **6E**).

The magnitude of inhibitory responses (Fig. **6F**) depended on the event considered (2-way Repeated Measures ANOVA, Event effect, F(2,181) = 12.44, *P =* 8.688x10^-6^) and the use of deconvolution (Deconvolution effect, F(1,181) = 21.72, *P =* 6.09x10^-6^) but we found no significant interaction between them (Event x Deconvolution, F(2,181) = 0.53, *P =* 0.585). Inhibitions to the stimulus were larger than those to the lever-presses and rewards (Bonferroni test on Event effect, *P =* 1.9x10^-5^ and *P =* 1.3x10^-5^, respectively), but lever-press and reward-evoked excitations did not differ from each other.

Together, these results indicate that Pf neurons respond strongly to reward-predictive stimuli but are also modulated in anticipation of lever-pressing and reward collection.

### 5.4 Modulation of Stimulus-evoked Responses by the Motivational State

**Fig. (7):**
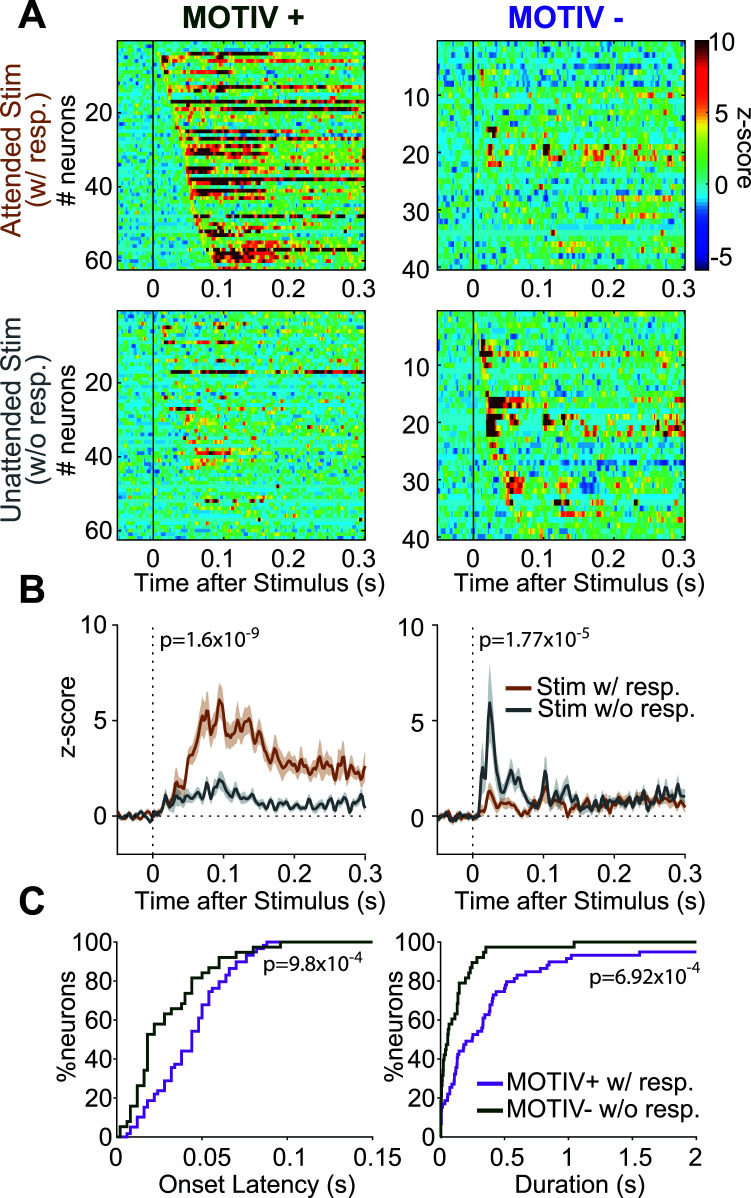
Pf neuronal excitations to the stimulus depend on whether the animal engages in reward-seeking. (**A**) Heatmaps showing stimuli-evoked excitations of neurons on trials of the animals engaged in reward-seeking (*top*) and those they did not (*bottom*) for MOTIV+ (*left) and MOTIV- (right*) neurons. Neurons are sorted by onset latencies. Raw PSTHs are plotted with 2 ms-time bins. (**B**) Average PSTHs for excited MOTIV + and excited MOTIV- neurons for attended (brown) and unattended (gray) stimuli. (**C**) Cumulative percentage of excitation latencies (*left)* and durations (*right*) for excited MOTIV+ (green) and excited MOTIV- (purple) neurons.

Our data showed that Pf neurons are strongly activated by stimuli when the rats engage in reward-seeking. We then sought to decipher whether stimuli-evoked neuronal modulations depended on motivational state. We took advantage of the fact that long sessions produce enough trials in which the rats engaged themselves in reward-seeking in response to the stimulus (attended trials) and those in which they did not (unattended trials, Fig. **7**). Visual inspection of the data revealed very brisk excitations leading us to use a higher time resolution (2 ms) to construct PSTHs and a shorter response duration requirement to detect excitations (4 ms). Because of the short latency of these responses that most certainly preceded the initiation of locomotion in response to the stimulus [36], we analyzed neuronal activity on raw and not deconvolved PSTHs. We observed a first population of 62 neurons (15.5%) with higher phasic activations in response to attended compared to unattended stimuli (2.87+/-0.37 and 0.99+/-0.20 Hz, respectively, paired t-test, t_61_ = 7.09, *P =* 1.63x10^-9^, Figs. **7A**, **B**). We named these neurons MOTIV+ because their activation of the stimulus reflects the animal's motivation to engage in the task. We also found a second population of 40 neurons (10%) that displayed an opposite pattern: they were more excited by unattended compared to attended stimuli (MOTIV- neurons, 0.52+/-0.13 and 1.75+/-0.33 Hz, respectively, paired t-test, t(39) = -4.89, *P =* 1.77x10^-5^, Figs. **7A**, **B**). The remaining neurons did not respond differently to stimuli on attended and unattended trials and are not presented here.

The analysis of the response profile dynamics revealed that excited MOTIV- neurons were excited at a considerably shorter onset latency than MOTIV+ neurons (30.4+/-3.6 ms and 44.2+/-2.8 ms, respectively, KS = 0.394, *P =* 9.81x10^-4^, Fig. **7C**). The excitations of excited MOTIV- neurons to unattended stimuli were also considerably shorter than those of excited MOTIV+ neurons to attended stimuli (117+/-29 ms and 436+/-87 ms, respectively, KS = 0.403, *P =* 6.92x10^-4^, Fig. **7C**).

The analysis of the basic electrophysiological features of this population (Supplementary Fig. **S1**) revealed that excited MOTIV+ neurons had a lower basal firing rate than excited MOTIV- neurons (8.59+/-1.55 and 19.4+/-3.27 Hz, respectively, KS = 0.36, *P =* 0.002) and a trend toward a longer spike width (223+/-14 and 186+/-10 ms, respectively, KS = 0.26, *P =* 0.056). We found no differences in the maximal frequency (146+/-16 and 151+/-15 Hz, respectively, KS = 0.22, *P =* 0.15) and the coefficient of variation (1.41+/-0.07 and 1.34+/-0.07, respectively, KS = 0.18, *P =* 0.34).

Within the population of excited MOTIV+ neurons, 66% were also excited by lever-presses, a proportion that was higher than that of the entire population (KS = 11.77, *P =* 0.003, Supplementary Fig. **S3**). Reward excitations were under-represented (KS = 5.80, *P =* 0.0055). For excited MOTIV- neurons, the overall responses to lever-presses and rewards were small and did not differ in proportion compared to the entire population (KS = 2.56, *P =* 0.28 and KS = 5.43, *P =* 0.07, Supplementary Fig. **S4**).

**Fig. (8):**
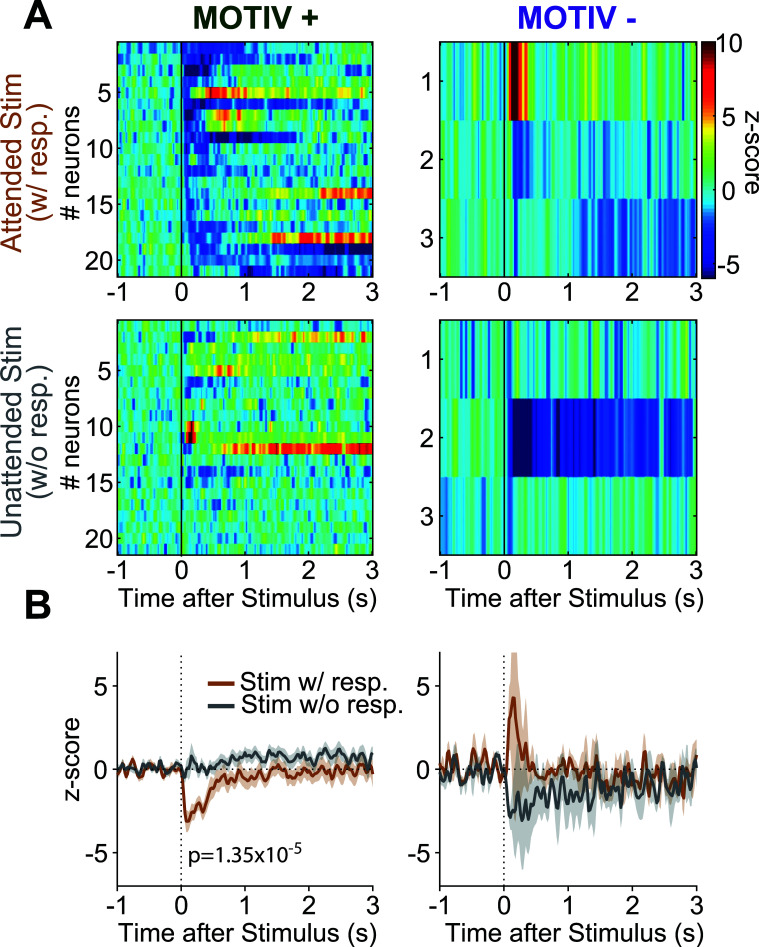
Pf neuronal inhibitions to the stimulus depend on whether the animal engages in reward-seeking. (**A**) Heatmaps showing stimuli-evoked inhibitions of neurons on trials of the animals engaged in reward-seeking (*top*) and those they did not (*bottom*) for MOTIV+ (*left) and MOTIV- (right*) neurons. Neurons are sorted by onset latencies. Raw PSTHs are plotted with 20 ms time bins. (**B**) Average PSTHs for inhibited MOTIV + and inhibited MOTIV- neurons for attended (brown) and unattended (gray) stimuli.

We conducted a similar analysis on stimuli-inhibited neurons on 20 ms-time-based PSTHs (Fig. **8**) and found 21 MOTIV+ and 3 MOTIV- neurons. Inhibited MOTIV+ neurons were more inhibited from attended than unattended stimuli (2.44+/-0.38 and 0.16+/-0.41 Hz, respectively, paired t-test, t(20) = -5.718, *P =* 1.35x10^-5^). The onset latency to attended stimuli was 78+/-11 ms and lasted 535+/-132 ms. Given the low number of inhibited MOTIV- neurons, we did not analyze them further.

Inhibited MOTIV+ neurons had a high basal firing rate of 25.66+/-4.3 Hz, a short spike width of 149+/-9 ms, a maximal frequency of 167+/-22 Hz and a coefficient of variation of 1.08+/-0.12 (Supplementary Fig. **S2**).

This population of neurons also had more inhibitory responses to the lever-press and the reward delivery than that found in the entire population (KS = 10.81, *P =* 0.004 and KS = 7.10, *P =* 0.028, supplementary Fig. **S5**).

## 6 DISCUSSION

We sought to characterize the electrophysiological activity of Pf neurons in rats performing a stimulus-driven reward-seeking task. Most Pf neurons respond to different task events with a higher proportion of excitations than inhibitions. The waveforms and discharge properties were not predictive of their excited or inhibited responses to task events. Stimuli evoked larger responses than lever-pressing and rewards collection. The most striking finding was that excitations to stimuli depended on whether the animal subsequently engaged in reward-seeking.

### 6.1 Basic Electrophysiological Properties of Pf Neurons

Pf neurons have traditionally been identified as a homogeneous population of glutamatergic neurons with long-range projections [23, 37, 38]. However, several studies have now revealed Pf subtypes with distinct morphological and electrophysiological signatures. Neurons with bushy dendritic trees have higher maximal discharge rates and burstiness than neurons with diffuse branching dendrites [39, 40]. Furthermore, a recent study found that in mice, the caudal Pf contains a small but significant proportion of GABA neurons that also have short action potential durations and high discharge rates [41]. Our analysis of the basic electrophysiologic features did reveal a strong dispersion in the parameters studied; they were strongly overlapping, and we could not determine criteria to cluster different populations. We did find that inhibited neurons had significantly higher basal firing rates and shorter spike widths, suggesting that they could correspond to GABA and/or bushy neurons. However, further studies are required to test whether different subtypes can be identified with parameters sampled in extracellular recordings.

### 6.2 Pf Neurons are Activated During the Behavioral Approach

We found a very high prevalence of neurons modulated by task events with a strong bias toward excitations. Stimuli clearly evoked the strongest responses for both excited and inhibited neurons. We speculate that this is related to the temporal unpredictability of stimuli presentations and their association with rewards that have both been shown to potentiate excitatory responses in primate CM/Pf [16, 28]. We found that stimuli-evoked responses occurred at a relatively constant latency with large variations in their duration. In many cases, excitations persisted until the rat lever-pressed. The long sessions allowed animals to demonstrate differences in task engagement. These differences were associated with large variability in the response latency within and between sessions, which likely explained the variability in the durations of excitations. Deconvolution allowed us to isolate neuronal activity time-locked to particular behavioral events by removing the contribution of neighboring events [34, 35]. The fact that deconvolution shortened stimulus- and action-evoked excitations suggest that the behavioral approach by itself activated Pf neurons. Interestingly, such a pattern of anticipatory activity seems to be found in both primates and mice [19, 31]. In a task where monkeys had to perform specific actions in response to instructive stimuli, long-latency facilitation (LLF) neurons recorded in CM displayed a biphasic response with inhibition followed by excitation to stimuli. The latter appeared more strongly correlated with the timing of the required action than the instructive stimulus directing it [31]. Furthermore, a local inactivation of the CM with the GABA agonist muscimol decreased the number of licks in a Pavlovian task [16], indicating that LLF activity contributes to behavioral responding. In mice performing a fixed-ratio 8 schedule task in which no explicit stimuli were presented, Pf neurons also displayed excitation that preceded the first lever-press, and that was shown to be necessary for task performance. Indeed, optogenetic inhibition of striatum-projecting Pf neurons increased the latency to re-engage in a trial without affecting the repeated motor sequence of lever-pressing [19]. Altogether, these results indicate that the excitation of Pf neurons during the behavioral approach is causal.

Amongst stimulus-inhibited neurons, a few (5 out of 399) displayed the characteristic biphasic inhibition-excitation found in LLF neurons, revealing a similarity with the primate CM. Inhibitions evoked by instrumental actions, often associated with preceding excitations, are also found in mice [19]. But overall, inhibitory responses were considerably less frequent than excitation. Thus, exploring their role will require further studies manipulating specifically these populations.

### 6.3 Influence of the Motivational State on Pf Responses to Incentive Stimuli

In primates, CM neurons respond to unconditioned auditory and visual stimulations [16]. The amplitude of these responses decreased as the stimuli became predictive of rewards, indicating that conditioning induced a form of long-term plasticity. However, whether Pf neuronal responses could be rapidly modulated was unknown. The use of long sessions allowed us to analyze Pf neuronal activity in response to stimuli predicting the same rewards while rats were in different motivational states. We compared the trials in which the animal attended to the stimulus by engaging in behavioral response (and thus obtaining the reward) and those they did not. The absence of engagement on unattended trials was unlikely due to a failure in perceiving the stimulus that was highly salient and long-lasting (300 ms-long 85 dB white noise coupled to the visual extension of the lever for 10 s). The strongest evidence against this hypothesis is the fact that we identified a population of neurons that was more excited about the stimulus on unattended trials, indicative of an active process taking place in these situations. Most likely, the absence of responding was caused by the fact that, at certain times, animals in these long sessions valued reward-seeking less than other activities (*e.g*., grooming, exploring, and resting).

MOTIV+ neurons exhibited either excitation or inhibition to attended stimuli in the first 100 ms of their presentations. In a similar task, locomotion onset has been reported to start ~250 ms after a reward-predictive stimulus, indicating that the initial component of the responses of MOTIV+ neurons was not driven by movements themselves [36] but could instead participate in initiating them. It seems unlikely that excited MOTIV+ neurons are the homolog to primate LLF neurons for two reasons. First, inhibition precedes the excitation in LLF neurons [16]. Second, the amplitude of the excitatory component of LLF neurons is inversely correlated with the reaction time on a trial-to-trial basis [31]. The stronger response to attended stimuli compared to unattended stimuli in the present study is diametrically opposed to this observation. Thus, the excited MOTIV+ profile reported in this study does not seem to match the LLF activity reported in primates. However, we did find, among inhibited MOTIV+ neurons, some profiles that matched primate LLF neurons. Both excited and inhibited MOTIV+ neurons could participate in action engagement in response to the stimulus. Testing this hypothesis will require tools to selectively manipulate these populations.

Excited MOTIV- neurons had stereotyped responses with excitations starting as early as 6 ms after stimulus onset and lasting less than 100 ms. Yet, these brisk responses were associated with the absence of behavioral responses in the next 10 seconds. These data provide further evidence that the Pf carries an important attentional function [10, 24, 28, 42] by gathering low-level sensory information that may arise from the deep layers of the superior colliculi and/or the pedunculopontine nucleus [43-45]. Excited MOTIV- neurons shared properties with short-latency facilitation neurons (SLF) recorded in the primate CM [16]. But importantly, the early and transient firing of excited MOTIV- neurons, manifested when the rat did not attend to stimuli, indicates that their attention was not directed toward them. On the contrary, CM recordings and inactivations in monkeys during a countermanding task provided evidence that SLF neurons participate in the direction of attention toward stimuli [28]. This apparent discrepancy could relate to the modality used in these studies (auditory and visual in our study and purely visual in the primate study) or even different functions carried by Pf neurons in different species. Another intriguing possibility lies in the characteristics of the stimuli used. Minamimoto and Kimura (2002) presented temporally predictable stimuli that provided instructions to the monkeys about the direction of the saccade to be rewarded. In our study, we used stimuli that were presented unexpectedly and incentivized the rats to switch from their current activity to reward-seeking by engaging in actions during a 10 s-time window. These actions strongly differed depending on the location of the rats at the time of occurrence of these stimuli. Thus, the Pf could subserve different roles for instructive and incentive stimuli, as we previously showed for NAc neurons [33].

The excitations of MOTIV- neurons could block processes that facilitate task engagement through the dense Pf projection to the striatal complex [5]. As opposed to other thalamic nuclei, the Pf preferentially synapses on cholinergic interneurons (CINs) [14, 46-48] that exert a strong inhibitory control on the activity of medium spiny projection neurons (MSNs) *in vivo* [49] through different GABA interneuron subtypes [13, 50, 51]. We recently reported that NAc Core CINs were also more active in response to unattended than attended incentive stimuli [33]. The short latencies observed in CINs suggest that they could be driven by excited MOTIV- Pf neurons.

Excitations of NAc MSNs evoked by incentive stimuli are necessary for rats to engage in action [35] and depend on the ventral tegmental area [52], basolateral amygdala [53] and paraventricular thalamus [20] inputs.

## CONCLUSION

The present work indicates that the Pf projection to NAc could subserve an opposite role by repressing behavioral responding. Further work is needed to directly test this hypothesis.

## Data Availability

Not applicable.

## LIST OF ABBREVIATIONS

CINs: Cholinergic Interneurons
CM: Center Médian
MSNs: Medium Spiny Projection Neurons
Pf: Parafascicular
PSTHs: Peri-stimulus Time Histograms
