# Supplementary material for: Two Distinct Neuronal Populations in the Rat Parafascicular Nucleus Oppositely Encode the Engagement in Stimulus-driven Reward-seeking
Source: Curr Neuropharmacol. 2024 Feb 1;22(9):1551–65. doi: 10.2174/1570159X22666240131114225 (PMC11097993; doi:10.2174/1570159X22666240131114225)
Supplement: Supplementary file 1 [file CN-22-1551_SD1.pdf]

Supplementary Material

Two Distinct Neuronal Populations in the Rat Parafascicular Nucleus Oppositely Encode the Engagement in Stimulus-driven Reward-seeking

Mehdi Sicre[1], Frederic Ambroggi[1,2,#] and Julie Meffre[1,#,*]

[1]Aix-Marseille Université, CNRS, Laboratoire de Neurosciences Cognitives, UMR 7291, Marseille, France; [2]Institut de Neurosciences de la Timone, Aix-Marseille Univ, CNRS, INT, Marseille, France

Table S1. Percentage and number (in parenthesis) of the different neuronal responses for individual rats.

			Excited			Inhibited	
Rat	n	Stimulus	Lever-Press	Reward	Stimulus	Lever-Press	Reward
1	**139**	39,6% (55)	53,2% (74)	52,5% (73)	5% (7)	18,7% (26)	21,6% (30)
2	**65**	20% (13)	53,8% (35)	50,8% (33)	10,8% (7)	30,8% (20)	27,7% (18)
3	**66**	53% (35)	60,6% (40)	59,1% (39)	4,5% (3)	9,1% (6)	6,1% (4)
4	**51**	56,9% (29)	66,7% (34)	68,6% (35)	2% (1)	21,6% (11)	21,6% (11)
5	**14**	28,6% (4)	50% (7)	57,1% (8)	14,3% (2)	42,9% (6)	28,6% (4)
6	**4**	75% (3)	75% (3)	75% (3)	0% (0)	25% (1)	0% (0)
7	**60**	26,7% (16)	60% (36)	61,7% (37)	6,7% (4)	20% (12)	18,3% (11)
Total	**399**	**155**	**229**	**228**	**24**	**82**	**78**

1875-6190/24 $65.00+.00 **© 2024 Bentham Science Publishers**

Table 2S. Percentage and number (in parenthesis) of the different neuronal response types for the stimulus, lever-press and reward delivery for raw and deconvolved data.

Stimulus	Lever-Press	Reward	Raw	Deconv.
Inhibited	Inhibited	Inhibited	3,8% (15)	1,8% (7)
Inhibited	Inhibited	No Resp.	0,3% (1)	0,8% (3)
Inhibited	Inhibited	Excited	0,5% (2)	0,5% (2)
Inhibited	No Resp.	Inhibited	0% (0)	0,3% (1)
Inhibited	No Resp.	No Resp.	0% (0)	1% (4)
Inhibited	No Resp.	Excited	0% (0)	0% (0)
Inhibited	Excited	Inhibited	0,3% (1)	0,8% (3)
Inhibited	Excited	No Resp.	0% (0)	0,8% (3)
Inhibited	Excited	Excited	1,3% (5)	2% (8)
No Resp.	Inhibited	Inhibited	7,8% (31)	4,8% (19)
No Resp.	Inhibited	No Resp.	1,3% (5)	2,5% (10)
No Resp.	Inhibited	Excited	2,8% (11)	4,5% (18)
No Resp.	No Resp.	Inhibited	2,8% (11)	2% (8)
No Resp.	No Resp.	No Resp.	14,8% (59)	25,1% (100)
No Resp.	No Resp.	Excited	2,5% (10)	3,3% (13)
No Resp.	Excited	Inhibited	1,8% (7)	1,8% (7)
No Resp.	Excited	No Resp.	3,8% (15)	4,5% (18)
No Resp.	Excited	Excited	17,8% (71)	12% (48)
Excited	Inhibited	Inhibited	2,3% (9)	1,8% (7)
Excited	Inhibited	No Resp.	0% (0)	0,8% (3)
Excited	Inhibited	Excited	2% (8)	4,8% (19)
Excited	No Resp.	Inhibited	0,5% (2)	0,5% (2)
Excited	No Resp.	No Resp.	1% (4)	3% (12)
Excited	No Resp.	Excited	0,5% (2)	0,8% (3)
Excited	Excited	Inhibited	1% (4)	2% (8)
Excited	Excited	No Resp.	1,5% (6)	2,8% (11)
Excited	Excited	Excited	30,1% (120)	15,5% (62)

Table 3S. Percentage and number (in parenthesis) of excited/inhibited MOTIV+/- neurons for individual rats.

		**Excited**		**Inhibited**	
**Rat**	**n**	**MOTIV+**	**MOTIV-**	**MOTIV+**	**MOTIV-**
1	**139**	18% (25)	5% (7)	3,6% (5)	0,7% (1)
2	**65**	15,4% (10)	10,8% (7)	6,2% (4)	3,1% (2)
3	**66**	7,6% (5)	12,1% (8)	6,1% (4)	0% (0)
4	**51**	23,5% (12)	21,6% (11)	3,9% (2)	0% (0)
5	**14**	7,1% (1)	0% (0)	7,1% (1)	0% (0)
6	**4**	25% (1)	0% (0)	0% (0)	0% (0)
7	**60**	13,3% (8)	11,7% (7)	8,3% (5)	0% (0)
Total	**399**	**62**	**40**	**21**	**3**

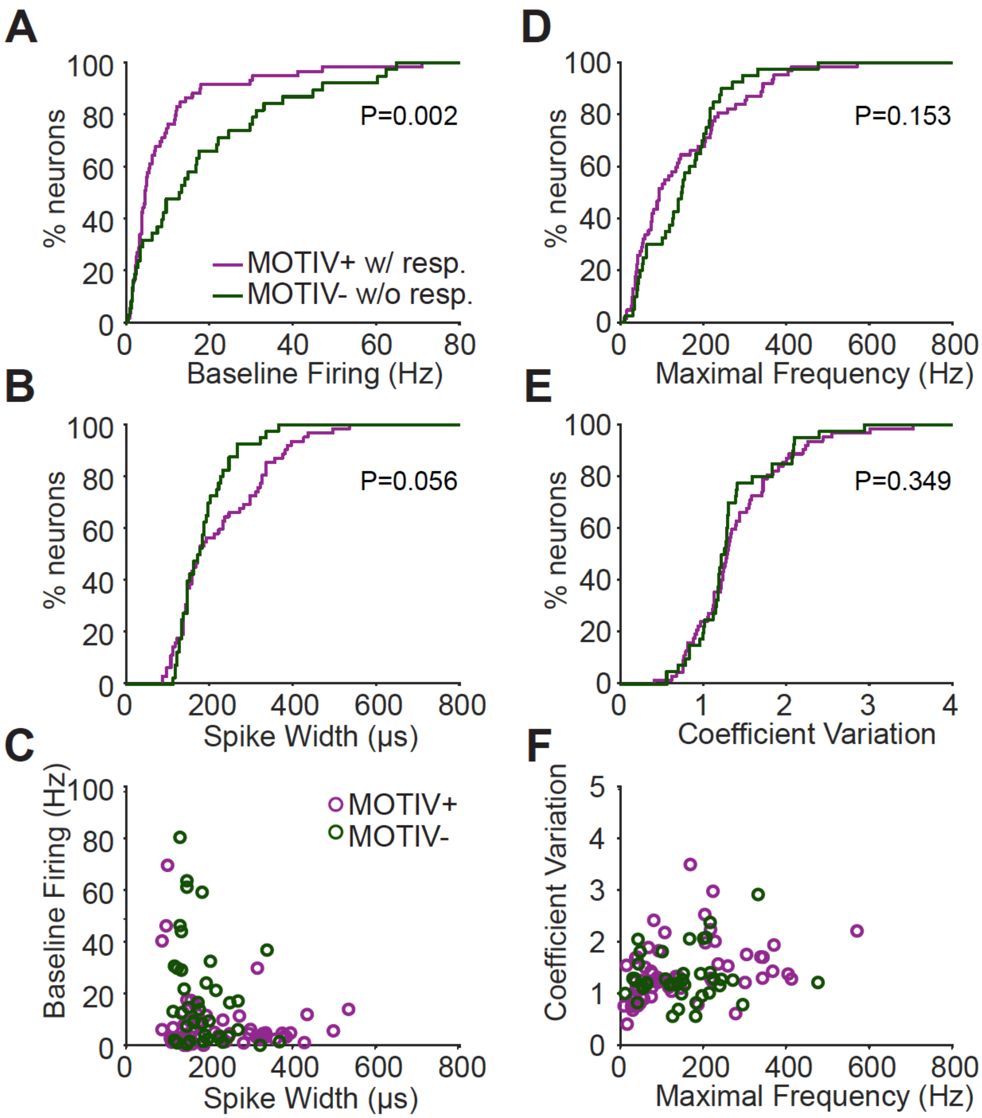

Supplementary Fig.1. Electrophysiological characteristics of excited MOTIV+ and MOTIV- neurons. **A.** Cumulative percentage of baseline firing rate of excited MOTIV+ (green) and excited MOTIV- (purple) neurons. **B.** Cumulative percentage of spike widths. **C.** Baseline firing rate plotted against spike width for individual neurons. **D.** Cumulative percentage of maximal frequencies. **E.** Cumulative percentage of the coefficients of variation. **H.** Coefficient of variations plotted against the maximal frequencies for individual neurons.

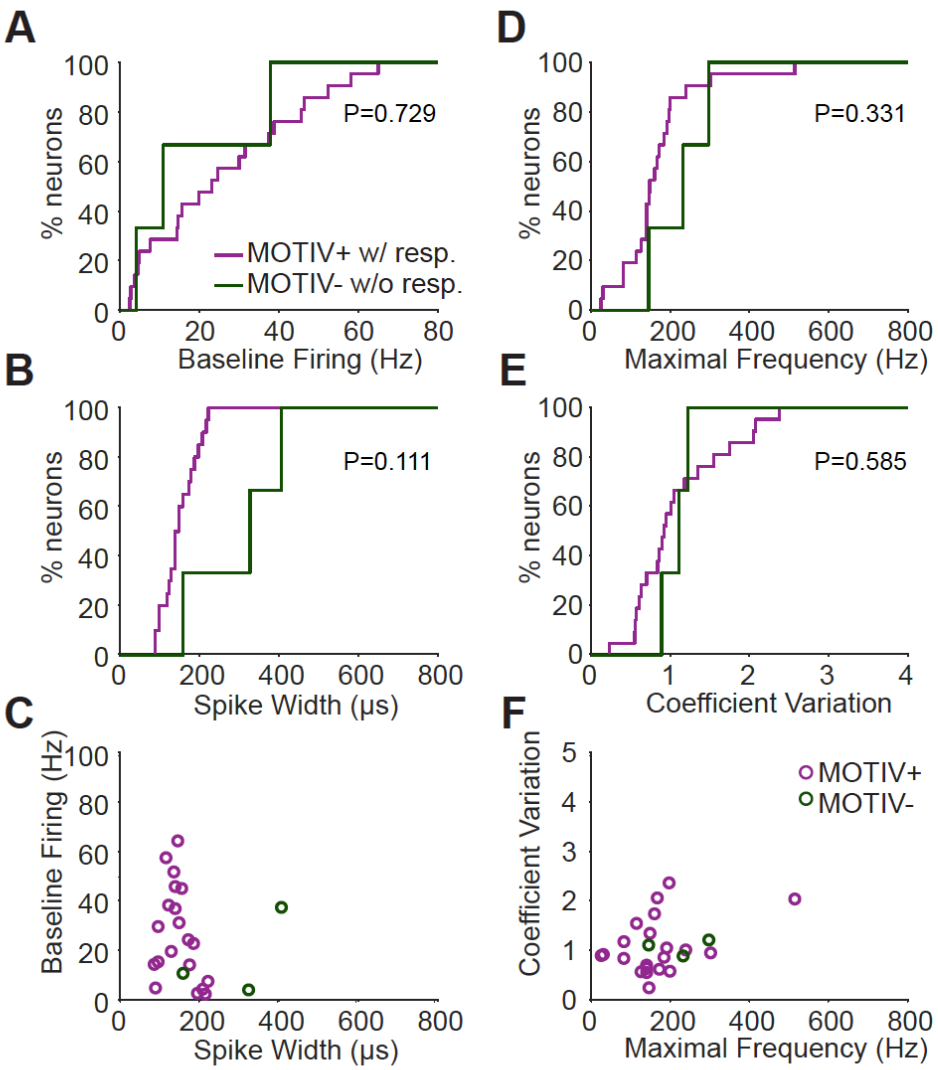

Supplementary Fig.2. Electrophysiological characteristics of inhibited MOTIV+ and MOTIV- neurons. A. Cumulative percentage of baseline firing rate of inhibited MOTIV+ (green) and inhibited MOTIV- (purple) neurons. **B.** Cumulative percentage of spike widths. **C.** Baseline firing rate plotted against spike width for individual neurons. **D.** Cumulative percentage of maximal frequencies. **E.** Cumulative percentage of the coefficients of variation. **H.** Coefficient of variations plotted against the maximal frequencies for individual neurons.

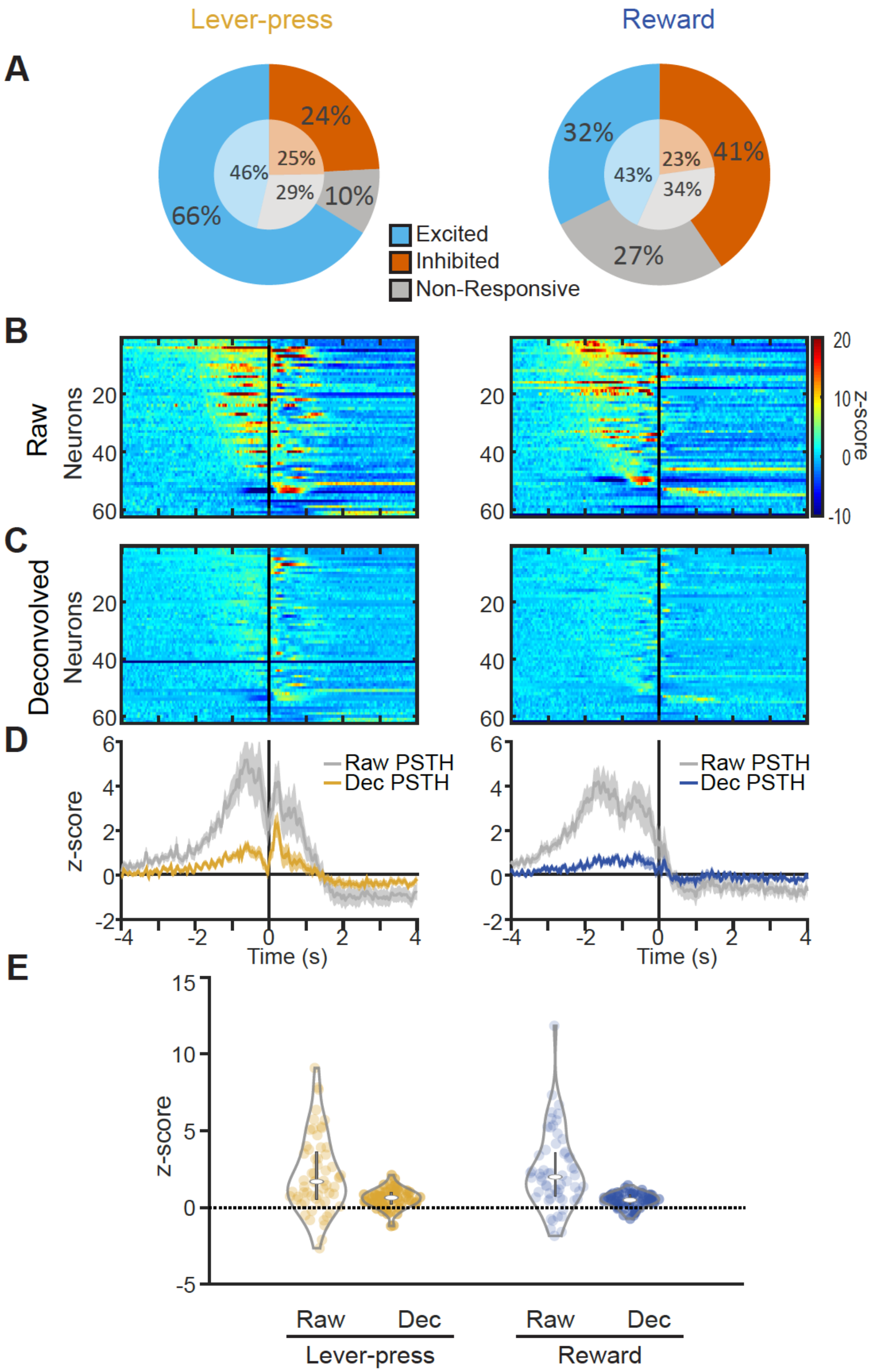

Supplementary Fig.3. Neuronal responses of Excited MOTIV+ neurons to lever-presses and reward deliveries. A. Proportion of excited, inhibited and non-responsive neurons for lever-presses (left) and reward deliveries (right). The internal pie charts represent the proportions of responses in the entire data set (n=399). The ring charts represent the same proportions for the population of excited MOTIV+ neurons. **B.** Heatmaps (top) and PSTHs (bottom). **C.** Violin plots of lever-press- and reward delivery-evoked firing.

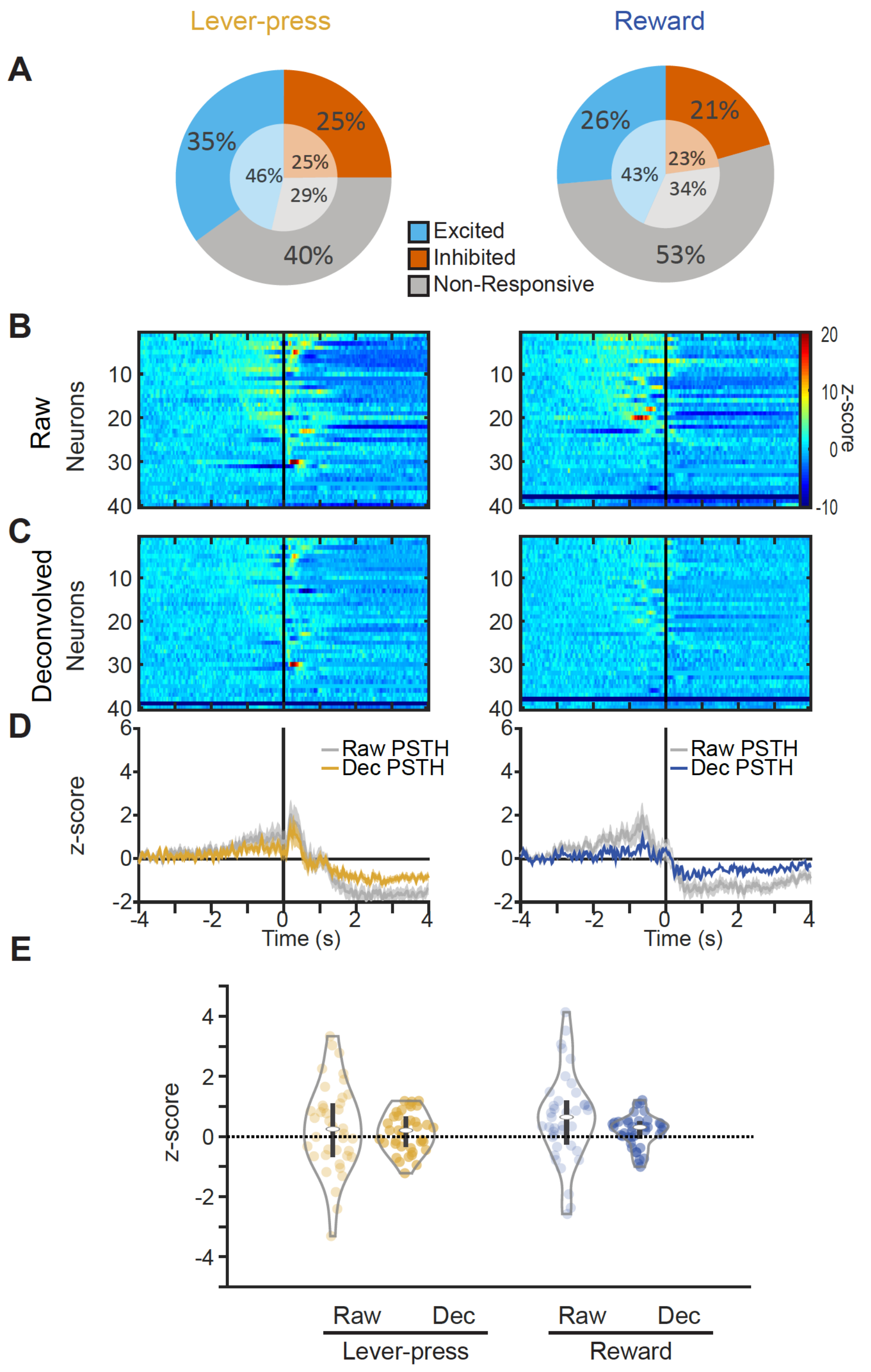

Supplementary Fig.4. Neuronal responses of excited MOTIV- neurons to lever-presses and reward deliveries. A. *Proportion of* excited, inhibited and non-responsive neurons for lever-presses (left) and reward deliveries (right). The internal pie charts represent the proportions of responses in the entire data set (n=399). The ring charts represent the same proportions for the population of excited MOTIV- neurons. **B.** Heatmaps (top) and PSTHs (bottom). **C.** Violin plots of lever-press- and reward delivery-evoked firing.

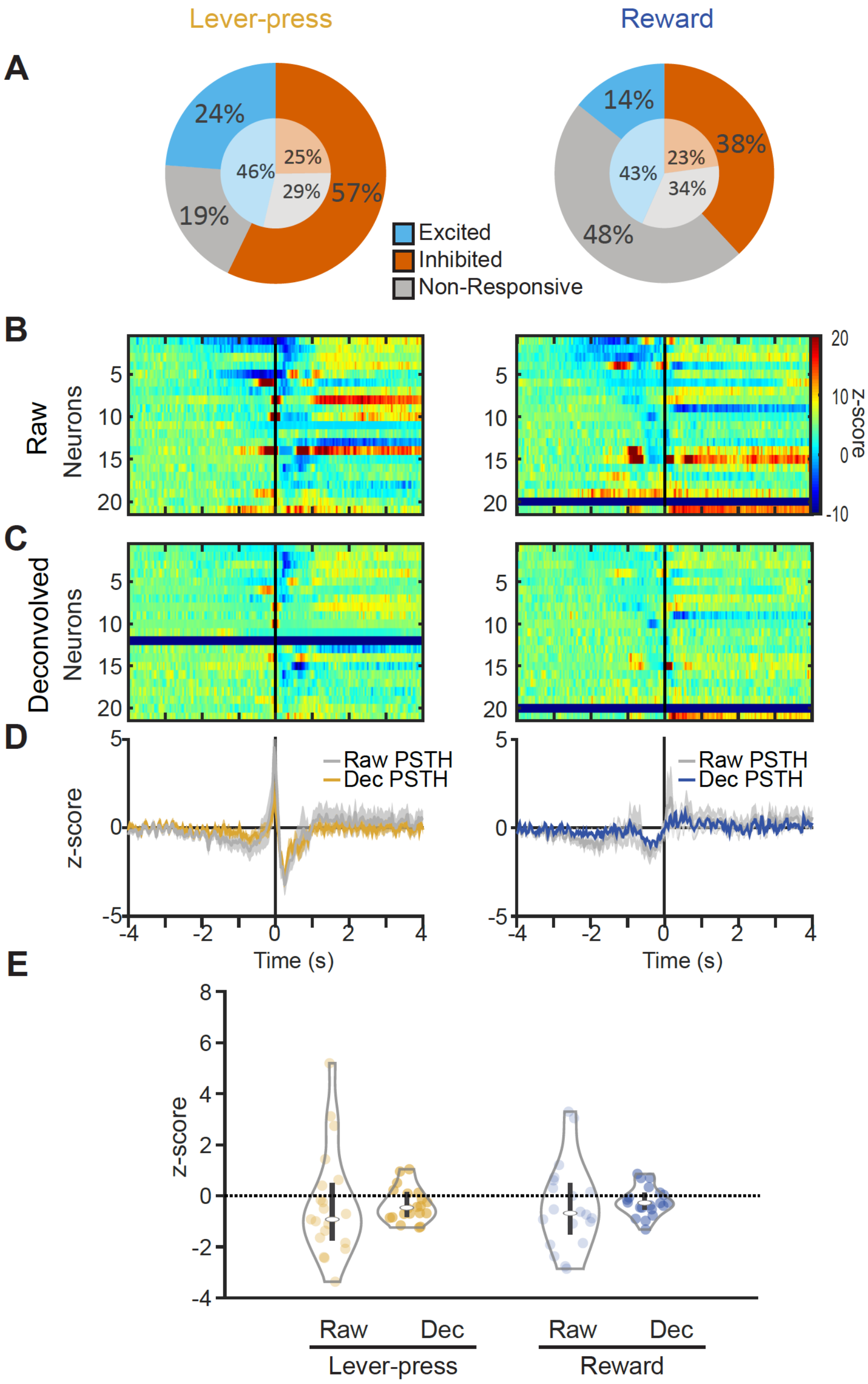

Supplementary Fig.5. Neuronal responses of inhibited MOTIV+ neurons to lever-presses and reward deliveries. A. *Proportion of* excited, inhibited and non-responsive neurons for lever-presses (left) and reward deliveries (right). The internal pie charts represent the proportions of responses in the entire data set (n=399). The ring charts represent the same proportions for the population of inhibited MOTIV+ neurons. **B.** Heatmaps (top) and PSTHs (bottom). **C.** Violin plots of lever-press- and reward delivery-evoked firing.
